# Mouse Genome-Wide Association and Systems Genetics Identify *Asxl2* As a Regulator of Bone Mineral Density and Osteoclastogenesis

**DOI:** 10.1371/journal.pgen.1002038

**Published:** 2011-04-07

**Authors:** Charles R. Farber, Brian J. Bennett, Luz Orozco, Wei Zou, Ana Lira, Emrah Kostem, Hyun Min Kang, Nicholas Furlotte, Ani Berberyan, Anatole Ghazalpour, Jaijam Suwanwela, Thomas A. Drake, Eleazar Eskin, Q. Tian Wang, Steven L. Teitelbaum, Aldons J. Lusis

**Affiliations:** 1 Center for Public Health Genomics, University of Virginia, Charlottesville, Virginia, United States of America; 2 Department of Medicine, Division of Cardiovascular Medicine, University of Virginia, Charlottesville, Virginia, United States of America; 3 Department of Biochemistry and Molecular Genetics, University of Virginia, Charlottesville, Virginia, United States of America; 4 Department of Medicine, David Geffen School of Medicine, University of California Los Angeles, Los Angeles, California, United States of America; 5 Department of Pathology and Immunology, School of Medicine, Washington University in St. Louis, St. Louis, Missouri, United States of America; 6 Department of Computer Science, University of California Los Angeles, Los Angeles, California, United States of America; 7 Department of Oral Biology, David Geffen School of Medicine, University of California Los Angeles, Los Angeles, California, United States of America; 8 Department of Pathology and Laboratory Medicine, David Geffen School of Medicine, University of California Los Angeles, Los Angeles, California, United States of America; 9 Department of Biological Sciences, University of Illinois at Chicago, Chicago, Illinois, United States of America; 10 Department of Human Genetics, David Geffen School of Medicine, University of California Los Angeles, Los Angeles, California, United States of America; 11 Department of Microbiology, Immunology, and Molecular Genetics, University of California Los Angeles, Los Angeles, California, United States of America; Wellcome Trust Centre for Human Genetics, University of Oxford, United Kingdom

## Abstract

Significant advances have been made in the discovery of genes affecting bone mineral density (BMD); however, our understanding of its genetic basis remains incomplete. In the current study, genome-wide association (GWA) and co-expression network analysis were used in the recently described Hybrid Mouse Diversity Panel (HMDP) to identify and functionally characterize novel BMD genes. In the HMDP, a GWA of total body, spinal, and femoral BMD revealed four significant associations (−log10P>5.39) affecting at least one BMD trait on chromosomes (Chrs.) 7, 11, 12, and 17. The associations implicated a total of 163 genes with each association harboring between 14 and 112 genes. This list was reduced to 26 functional candidates by identifying those genes that were regulated by local eQTL in bone or harbored potentially functional non-synonymous (NS) SNPs. This analysis revealed that the most significant BMD SNP on Chr. 12 was a NS SNP in the *additional sex combs like-2* (*Asxl2*) gene that was predicted to be functional. The involvement of *Asxl2* in the regulation of bone mass was confirmed by the observation that *Asxl2* knockout mice had reduced BMD. To begin to unravel the mechanism through which *Asxl2* influenced BMD, a gene co-expression network was created using cortical bone gene expression microarray data from the HMDP strains. *Asxl2* was identified as a member of a co-expression module enriched for genes involved in the differentiation of myeloid cells. In bone, osteoclasts are bone-resorbing cells of myeloid origin, suggesting that *Asxl2* may play a role in osteoclast differentiation. In agreement, the knockdown of *Asxl2* in bone marrow macrophages impaired their ability to form osteoclasts. This study identifies a new regulator of BMD and osteoclastogenesis and highlights the power of GWA and systems genetics in the mouse for dissecting complex genetic traits.

## Author Summary

Osteoporosis is a disease of weak and fracture-prone bones. The characteristic of bone that is most predictive of fractures is low bone mineral density (BMD), a trait primarily controlled by genetics. In recent years, significant advances have been made in the discovery of genes affecting BMD; however, our understanding of its genetic basis is still primitive. In this study, we used genome-wide association in the mouse to identify *additional sex combs like-2* (*Asxl2*) as a novel BMD gene. In confirmation of our genetic analysis, mice deficient in *Asxl2* had reduced BMD. To evaluate its function in bone, the expression levels of *Asxl2* and tens of thousands of other genes were measured in bone in a large number of inbred mouse strains. *Asxl2* demonstrated a pattern of expression indicative of genes that play a critical role in osteoclasts, the cells that are responsible for bone resorption. Further study of *Asxl2* may reveal novel therapeutic targets for the treatment and prevention of osteoporosis.

## Introduction

Osteoporosis is a common disease characterized by bone fragility and an increased risk of fracture [1]. One of strongest predictors of fracture is low bone mineral density (BMD) [2] and while BMD is influenced by both genetic and environmental factors, most (between 60% and 80%) of its variance is heritable [3]. Thus, the identification of novel BMD genes is critical for the discovery of new pathways and gene networks that will advance our understanding of basic bone biology and identify new therapeutic targets with the potential to combat osteoporosis.

Due in large part to its many advantages, such as the ability to experimentally cross genetically defined strains and perturb candidate genes, the mouse has played an instrumental role in the genetic analysis of BMD [4]. However, progress has been limited by low-resolution linkage-based quantitative trait loci (QTL) mapping approaches and the difficulties inherent to QTL cloning [5]. As a result, mouse linkage approaches have lead to the identification of only three BMD quantitative trait genes, *Alox12* [6], *Sfrp4* [7] and *Darc* [8], even though hundreds of QTL have been mapped [9]. In part due to the success of genome-wide association (GWA) in humans, several groups have investigated the prospects of high-resolution association mapping approaches in the mouse. One of the main challenges facing GWA in the mouse has been determining the most ideal population for such analyses. To date, mouse GWA studies have been performed with varying success using small sets of classical laboratory strains [10], advanced intercross lines [11], heterogeneous stocks [12], outbred mice [13], [14] and the Hybrid Mouse Diversity Panel (HMDP) [15]. One of the most promising is the HMDP, a collection of ∼100 classical laboratory and recombinant inbred (RI) strains that have been genotyped at ∼135,000 SNPs [15]. The primary advantage of the HMDP is that mapping resolution is over an order of magnitude higher than with linkage. We have recently demonstrated through simulations that associations explaining 5% of the phenotypic variance in a trait have 95% confidence intervals (CIs) of ∼2.6 megabases (Mb) [15]. This is in comparison to CIs for mouse linkage studies that are typically in the range of 40–60 Mb [13]. Additionally, statistical power in the HDMP has been found to be adequate to map variants affecting complex traits [15]. Moreover, phenotypes can be mapped in the HMDP without the need for costly genotyping and one can collect multiple specimens (e.g. tissues, individual cell-types, etc.) from strains for molecular profiling that are difficult or impossible to collect from a single mouse [15].

A drawback of both mouse and human GWA studies (and all “genotype to phenotype” mapping approaches) is their inability to provide information on how associated genes actually influence disease [16]. In many cases, it takes years to decipher the underlying biology of novel gene discoveries. One way to begin to provide functional information is through the use of systems genetics [17]. Systems genetics is an approach that incorporates molecular phenotypes, most commonly gene expression microarray profiles, into the genetic analysis of clinical phenotypes. One way that systems genetics can be used to functionally annotate genes of unknown function is through the generation of gene co-expression networks. Co-expression networks are created by clustering genes based on patterns of co-expression across a series of perturbations, such as the differing genetic backgrounds in the HMDP [18]. Co-expressed gene clusters or “modules” have been shown to be enriched for genes involved in the same general function [19], [20], [21], allowing one to annotate genes through “guilt-by-association” [22]. For example, if an uncharacterized gene is co-expressed with genes known to be involved in a biological process such as “apoptosis”, then it is more likely than not the unknown gene is also involved in “apoptosis”. The use of network analysis of systems genetic data can help to inform GWA discoveries by providing clues as to a gene's function in a physiologically-relevant context.

The goal of the current study was to identify and functionally characterize novel BMD genes using GWA and systems genetics in the HMDP. This approach implicated *additional sex combs like-2* (*Asxl2*) as the gene responsible for a BMD association on chromosome (Chr.) 12. This was further strengthened by the observation that *Asxl2* knockout mice had reduced BMD. Furthermore, gene co-expression analysis of bone transcriptomic data predicted that *Asxl2* was involved in the differentiation of bone-resorbing osteoclasts. In support of this prediction, osteoclastogenesis was impaired in bone marrow macrophages in which *Asxl2* expression was reduced by RNA interference. Together, these data are consistent with the hypothesis that *Asxl2* is a novel regulator of BMD and osteoclastogenesis.

## Results

### Genome-wide association analysis of BMD in the HMDP

**Figure 1:**
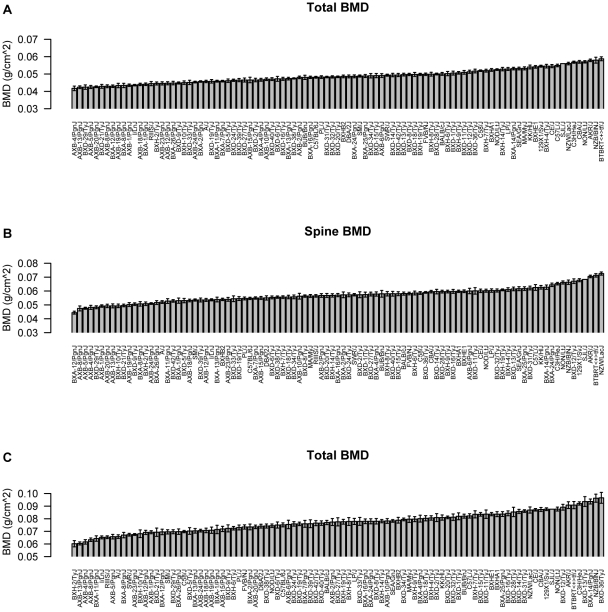
Characterization of bone mineral density (BMD) in the HMDP. Mean±SEM for (A) total body (TBMD), (B) spine (SBMD) and (C) femur (FBMD) BMD (g/cm^2^) in 97 HMDP inbred mouse strains. Differences between the strains with the lowest and highest values were 1.4, 1.6 and 1.6-fold for TBMD, SBMD and FBMD, respectively.

A detailed description of the HMDP, including strain selection and evaluation of statistical power and mapping resolution, is provided in [15]. Towards identifying genomic regions associated with BMD, we phenotyped 16-week old male mice (N = 879) from 97 HMDP strains (N = 9.1 mice/strain) for total body (TBMD), lumbar spine (SBMD) and femur (FBMD) areal BMD (Table S1). A wide range of BMD values were observed across the HMDP with differences of 1.4, 1.6 and 1.6-fold between the lowest and highest strains for TBMD, SBMD and FBMD, respectively (Figure 1).

**Figure 2:**
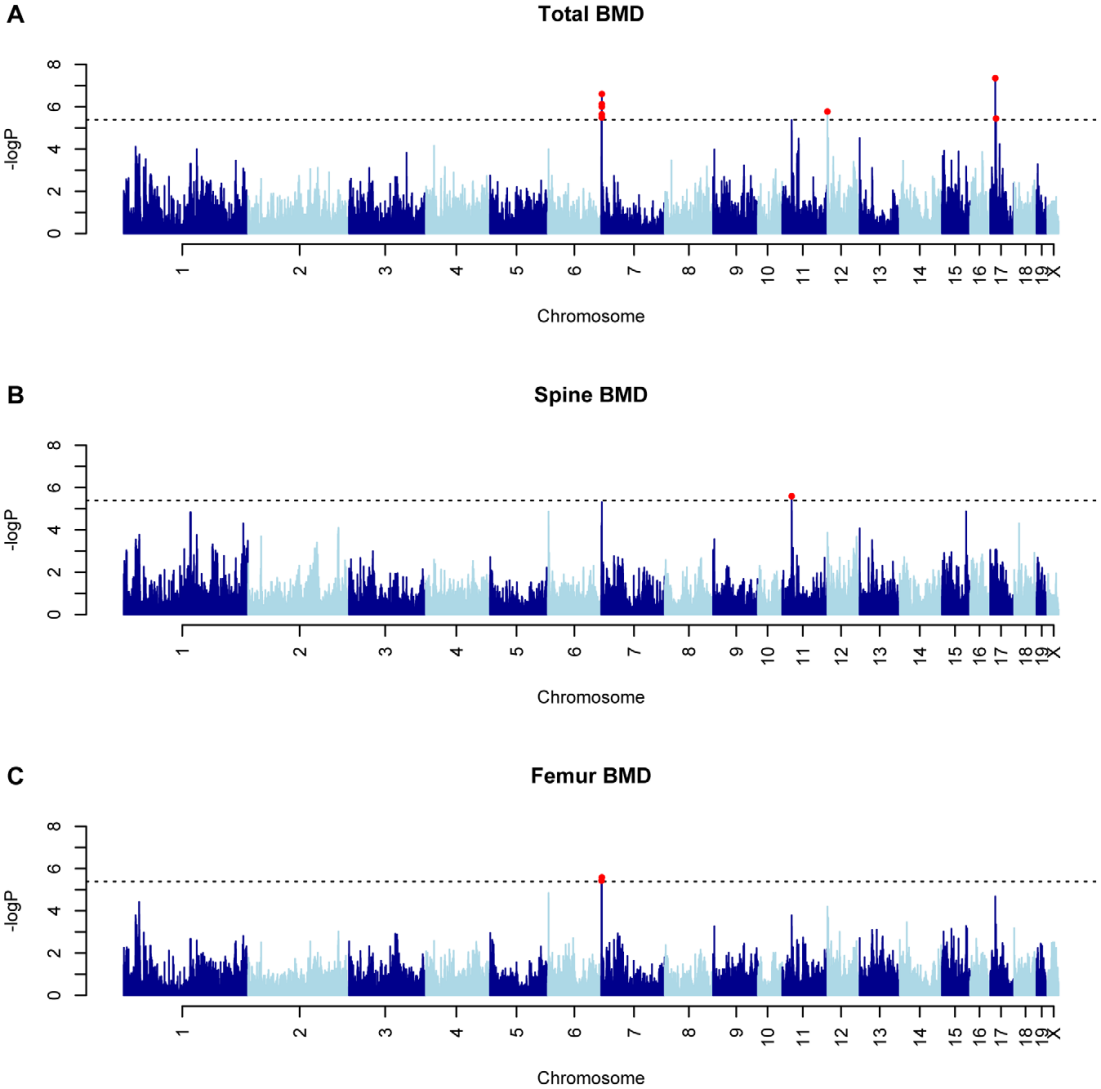
GWAS results for BMD in the HMDP. Manhattan plots showing the −log10 of the association P-values (−logP) for (A) total body (TBMD), (B) spine (SBMD) and (C) femur (FBMD) in 97 HMDP strains. The analysis was performed using 108,064 SNPs with a minor allele frequency >5%. Each mouse chromosome is plotted on the x-axis in alternating dark and light blue colors. SNPs in red on Chrs. 7, 12 and 17 for TBMD, Chr. 11 for SBMD and Chr. 7 for FBMD exceeded the predetermined genome-wide significance threshold of −logP = 5.39.

To identify associations for the three BMD phenotypes we used the Efficient Mixed-Model Association (EMMA) algorithm [23]. Adjusted association P-values were calculated for 108,064 SNPs with minor allele frequencies >5%. We have previously demonstrated that the P<0.05 genome-wide equivalent for GWA using EMMA in the HMDP is P = 4.1×10^−6^ (−log10P = 5.39) [23]. At this threshold, associations on chromosomes (Chrs.) 7, 12 and 17 were identified influencing TBMD (Figure 2A). A fourth unique association on Chr. 11 was identified for SBMD (Figure 2B) and the only significant locus for FBMD was the Chr. 7 association also affecting TBMD (Figure 2C). The details of each association are provided in Table 1. Importantly, each of these regions overlap with the location of QTL for areal BMD measures previously identified by linkage in F2 crosses [9].

**Table 1:** GWA results for BMD in the HMDP.

Trait^a^	Chr	SNP	Position (Mb)^b^	−logP	MAF^c^	95% CI (Mb)^b^		No. of genes^d^	Human Region (Chr: Start Mb – End Mb)
						Start	Stop		
TBMD	7	rs32149600	20.01	6.60	0.12	18.71	21.31	92	Chr19:49.7–51.2
FBMD	7	rs32149600	20.01	5.59	0.12	18.71	21.31	92	Chr19:49.7–51.2
SBMD	11	rs26963925	56.77	5.60	0.41	55.47	58.07	14	Chr5:151.3–154.3
TBMD	12	rs29131970	3.50	5.77	0.13	2.20	4.80	17	Chr2:24.2–26.2
TBMD	17	rs33294019	43.91	7.35	0.15	42.61	45.21	19	Chr6:44.9–48.0

**^a^**TBMD, total body BMD; FBMD, femur BMD; SBMD, spine BMD.

**^b^**Locations based on NCBI's Build37 genome assembly.

**^c^**MAF, minor allele frequency.

**^d^**Number of RefSeq genes located in the mouse association CI based on genome annotation from NCBI's Build37 assembly.

### Characterization of BMD associations

We have previously shown that the 95% confidence interval (CI) for the distribution of distances between the most significant and true causal SNPs, for simulated associations that explain 5% of the variance in the HMDP, is ∼2.6 Mb [24]. Therefore, we used this interval to conservatively define the boundaries of the four associations (Table 1). Within each association there were a total of 112 (Chr. 7), 14 (Chr. 11), 18 (Chr. 12) and 19 (Chr. 17) unique RefSeq genes (a full list of genes is provided in Table S2). We next identified those genes possessing functional alterations that might underlie the associations. Genes were selected based on whether they were regulated by a local expression QTL (eQTL) in the HMDP or if they harbored a non-synonymous (NS) SNP that was predicted to have functional consequences. For the eQTL analysis, we generated gene expression microarray profiles using RNA isolated from cortical bone in 95 of the 97 HMDP strains (N = 1–3/arrays per strain). EMMA was then used to perform an association analysis between all SNPs and array probes mapping within each region. A total of 74 genes were represented by at least one probe, after excluding probes that overlapped SNPs present among the classical inbred strains used in the HMDP (see Methods). Of these, 11 genes (8 within the Chr. 7 association and 3 within the Chr. 17 association) were identified with at least one probe whose expression was regulated by a significant (P≤5.1×10^−4^; Bonferroni corrected for the number of probes tested) local eQTL (Table 2 and data for all genes is provided in Table S2). In addition, we identified a total of 19 NS SNPs in 14 genes that were predicted to be either “Possibly Damaging” or “Probably Damaging” by PolyPhen [25], [26] (Table 3 and a list of all NS SNPs is provided in Table S3). A nonsense SNP was also identified in the meprin A, alpha (*Mep1a*) gene (Table 3). Therefore, of the 163 positional candidate genes, 26 were found to be regulated by a local eQTL in bone or harbored potentially functional NS SNPs. The number of functional candidate genes within each association was 12 (Chr. 7), 2 (Chr. 11), 3 (Chr. 12) and 9 (Chr. 17).

**Table 2:** Genes within BMD associations regulated by local eQTL in bone.

Gene	RefSeq	Chr	txStart (bp)^a^	txEnd (bp)^b^	Local eQTL P
*Pglyrp1*	NM_009402	7	19470038	19475787	1.3×10^−4^
*Irf2bp1*	NM_178757	7	19589413	19592112	3.9×10^−8^
*Dmwd*	NM_010058	7	19661548	19668124	1.8×10^−14^
*Six5*	NM_011383	7	19679892	19683694	6.5×10^−5^
*Ercc2*	NM_007949	7	19967387	19981041	2.7×10^−4^
*Bloc1s3*	NM_177692	7	20091152	20093680	2.5×10^−5^
*Trappc6a*	NM_025960	7	20094077	20101494	1.6×10^−7^
*Gemin7*	NM_027189	7	20150297	20158692	8.0×10^−6^
*Tnfrsf21*	NM_178589	17	43153503	43226137	1.6×10−^14^
*Cyp39a1*	NM_018887	17	43804373	43888380	4.6×10^−18^
*Rcan2*	NM_207649	17	43938799	44176465	1.3×10^−4^

**^a^**txStart, location of transcription start based on NCBI Build37 genome assembly.

**^b^**txEnd, location of transcription end based on NCBI Build37 genome assembly.

**Table 3:** Genes within BMD associations harboring non-synonymous SNPs predicted to alter protein function.

SNP	Chr	Location (bp)^a^	Alleles^b^	dbSNP_ann^c^	PolyPhen^d^	r^2^ e
rs32410945	7	19943262	C/G	Cd3eap:Cn:RG:89	Possibly Damaging	0.13
rs32422546	7	20288622	A/G	Tomm40:Cn:HY:204	Probably Damaging	0.31
rs13465077	7	20370636	A/G	Cblc:Cn:DG:415	Possibly Damaging	0.64
rs49286080	7	20555548	C/G	Ceacam20:Cn:HQ:57	Possibly Damaging	0.85
rs47432737	7	20555772	C/T	Ceacam20:Cn:TI:132	Possibly Damaging	0.85
rs46058112	7	20561620	G/T	Ceacam20:Cn:GC:406	Possibly Damaging	0.42
rs26966327	11	57953077	C/G	Gemin5:Cn:SC:807	Possibly Damaging	0.15
rs26950310	11	58001304	C/T	Irgb10:Cn:TM:14	Possibly Damaging	0.15
rs51508182	12	3501078	C/T	Asxl2:Cn:TI:939	Possibly Damaging	0.36
rs29131970	12	3501831	C/T	Asxl2:Cn:SF:1190	Probably Damaging	1.00
rs48727919	12	4216641	A/C	Cenpo:Cn:VF:155	Probably Damaging	0.09
rs48755392	12	4722485	C/T	4930417G10Rik:Cn:HR:256	Possibly Damaging	0.36
rs13482992	17	42803796	A/G	Gpr115:Cn:AV:534	Possibly Damaging	0.02
rs46389302	17	42846983	A/G	Gpr111:Cn:SF:632	Probably Damaging	0.00
rs51535437	17	43578895	A/T	Gpr116:Cn:DV:592	Probably Damaging	0.05
rs48412763	17	43628670	A/G	Mep1a:Cn:Q*:150	Nonsense	0.05
rs51295335	17	43739797	A/C	Pla2g7:Cn:ED:226	Probably Damaging	0.05
rs33536829	17	43761286	C/T	Tdrd6:Cn:YC:1939	Probably Damaging	0.01
rs49026999	17	4376341	C/T	Tdrd6:Cn:YC:1231	Possibly Damaging	0.05
rs45824990	17	43763779	C/T	Tdrd6:Cn:HR:1108	Probably Damaging	0.06

**^a^**SNP location based on NCBI Build37 genome assembly.

**^b^**Observed alleles.

**^c^**dbSNP annotation given as Gene:snp effect:amino acid change relative to observed alleles: position in protein sequence.

**^d^**Polyphen prediction.

**^e^**Linkage disequilibrium (r2) between each SNP and the peak BMD SNP within its respective region.

We also determined if any of the 163 genes implicated by GWA have been previously implicated in bone development. The associations on Chrs. 11 and 12 did not harbor known bone genes. In contrast, three (*Fosb* [27], *RelB* [28] and *Apoe* [29]) of the Chr. 7 genes have been linked to the regulation of bone mass. All three were located in close proximity to the association peak. *Fosb* was 124 kilobases (Kb) upstream, *Relb* was 180 Kb downstream and *Apoe* was 270 Kb downstream of rs32149600, the most significantly associated SNP on Chr. 7. Additionally, of the 19 Chr. 17 genes, *Runx2*, the “master regulator” of osteoblast differentiation [30], was located 1.0 Mbp downstream of rs33294019, the most significant SNP. None of the known bone genes were regulated by local bone eQTL or harbored potentially functional NS SNPs.

### Detailed analysis of the Chr. 12 association implicates *Asxl2* as a regulator of BMD

**Figure 3:**
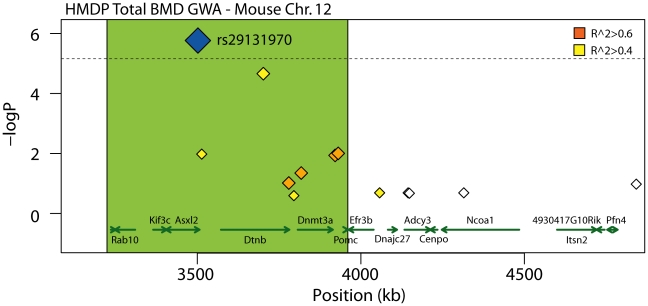
Close-up view of the TBMD Chr. 12 association in the HMDP. Distal segment (proximal region was truncated because it was the beginning of Chr. 12 and did not harbor any known or predicted genes) of the 2.6 Mb, 95% CI interval for the total body BMD (TBMD) association on mouse Chr. 12. The blue diamond represents the most significant SNP. SNPs are colored based on their LD with the most significant SNP; orange SNPs are in LD at r^2^>0.6, yellow SNPs are in LD at r^2^>0.4 and white SNPs are at r^2^<0.4. The green shaded box approximates the extent of moderate LD (r^2^>0.6) and outlines the most likely position of the causal gene. The positions of all RefSeq genes are plotted at the bottom using genome locatins from NCBI's Build37 genome assembly. Directional green arrows indicate transcript orientation.

All 26 of the genes identified above are candidates for the BMD associations and warrant further investigation. However, the goal of this analysis was to identify a gene(s) that was the most likely causal gene for an association. Due to Chr. 7 and Chr. 17 possessing multiple functional candidates (12 and 9, respectively), we could not identify the most likely candidate based on the existing data for either of the associations; therefore, we focused on the Chr. 11 and Chr. 12 associations due to the presence of only 2 and 3 functional candidates, respectively. All five of these genes harbored potentially functional NS SNPs. The expression of these genes was not regulated by local eQTL. Of the five, the additional sex-combs like 2 (*Asxl2*) was the most compelling candidate due to the fact that rs29131970, a NS SNP in *Asxl2* that was predicted to be functional, was also the peak SNP for Chr. 12 BMD association (Table 3 and Figure 3). Rs29131970 results in a phenylalanine (F) to serine (S) substitution at amino acid 1191 of the mouse ASXL2 protein. The mouse reference genome (the C57BL/6J strain) harbors the “C” allele, which codes for S; whereas, the rat, human, orangutan, dog, horse, opossum and chicken reference genomes all have the “T” allele, which codes for F. HMDP strains homozygous for the “C” allele had lower BMD relative to strains with the “T” allele (data not shown). A role for the other four candidates possessing NS SNPs on Chr. 11 and Chr. 12 appear to be less likely because of the modest linkage disequilibrium (LD) (r^2^ between 0.09 and 0.36) among the HMDP classical inbred strains between these SNPs and the SNPs most significantly associated with BMD for each region (Table 3). Therefore, based on the existing data we hypothesized that *Asxl2* was the causal gene for the Chr. 12 association.

### *Asxl2* knockout mice have reduced BMD

**Figure 4:**
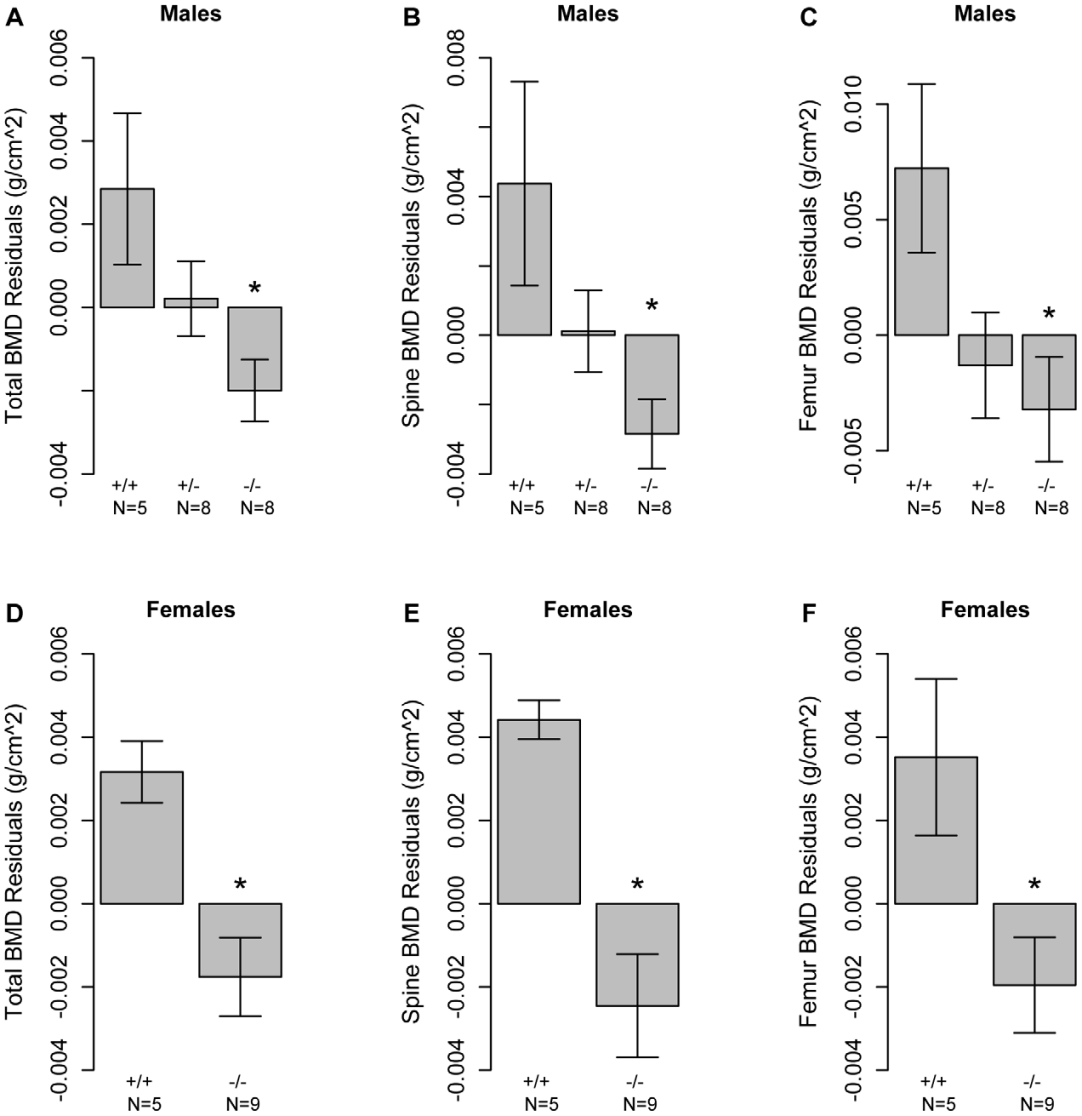
Mice deficient for *Asxl2* have decreased BMD. Male mice deficient in *Asxl2* (−/−) display significant decreases relative to wild-type controls (+/+) in (A) TBMD, (B) SBMD and (C) FBMD residuals after adjusted for age and body weight. (B) Female mice deficient in *Asxl2* (−/−) display significant decreases relative to wild-type controls (+/+) in (D) TBMD, (E) SBMD and (F) FBMD residuals after adjusted for age and body weight. Data shown in all panels are residual means±SEM. *P<0.05.

To directly test the hypothesis that *Asxl2* was involved in the regulation of BMD we characterized TBMD, SBMD and FBMD in *Asxl2* knockout mice (*Asxl2* ^−/−^). The mice used for this experiment were between the ages of 2–4 months and as previously reported [31] a significant (P<0.05) reduction in body weight in *Asxl2* ^−/−^ mice was observed (data not shown). To evaluate bone mass in the absence of these confounding effects we evaluated the BMD residuals across genotype after adjusting for age and body weight within each sex. This analysis revealed significant (P<0.05) reductions in TBMD, SBMD and FBMD residuals in male *Asxl2* ^−/−^ mice as compared to wild-type (*Asxl2^+/+^*) controls (Figure 4A–4C). In addition, male heterozygous (*Asxl2^+/−^*) mice demonstrated an intermediate phenotype for all BMD measures, although the differences were not statistically different from either homozygous genotype (Figure 4A–4C). We also observed similar decreases in TBMD, SBMD and FBMD residuals in female *Asxl2* ^−/−^ mice as compared to *Asxl2^+/+^* controls (Figure 4D–4F). These data confirm that *Asxl2* is a regulator of BMD and are consistent with the hypothesis that *Asxl2* is responsible for the genetic association on Chr. 12 in the HMDP.

### Transcriptional network analysis predicts that *Asxl2* is involved in osteoclastogenesis

**Figure 5:**
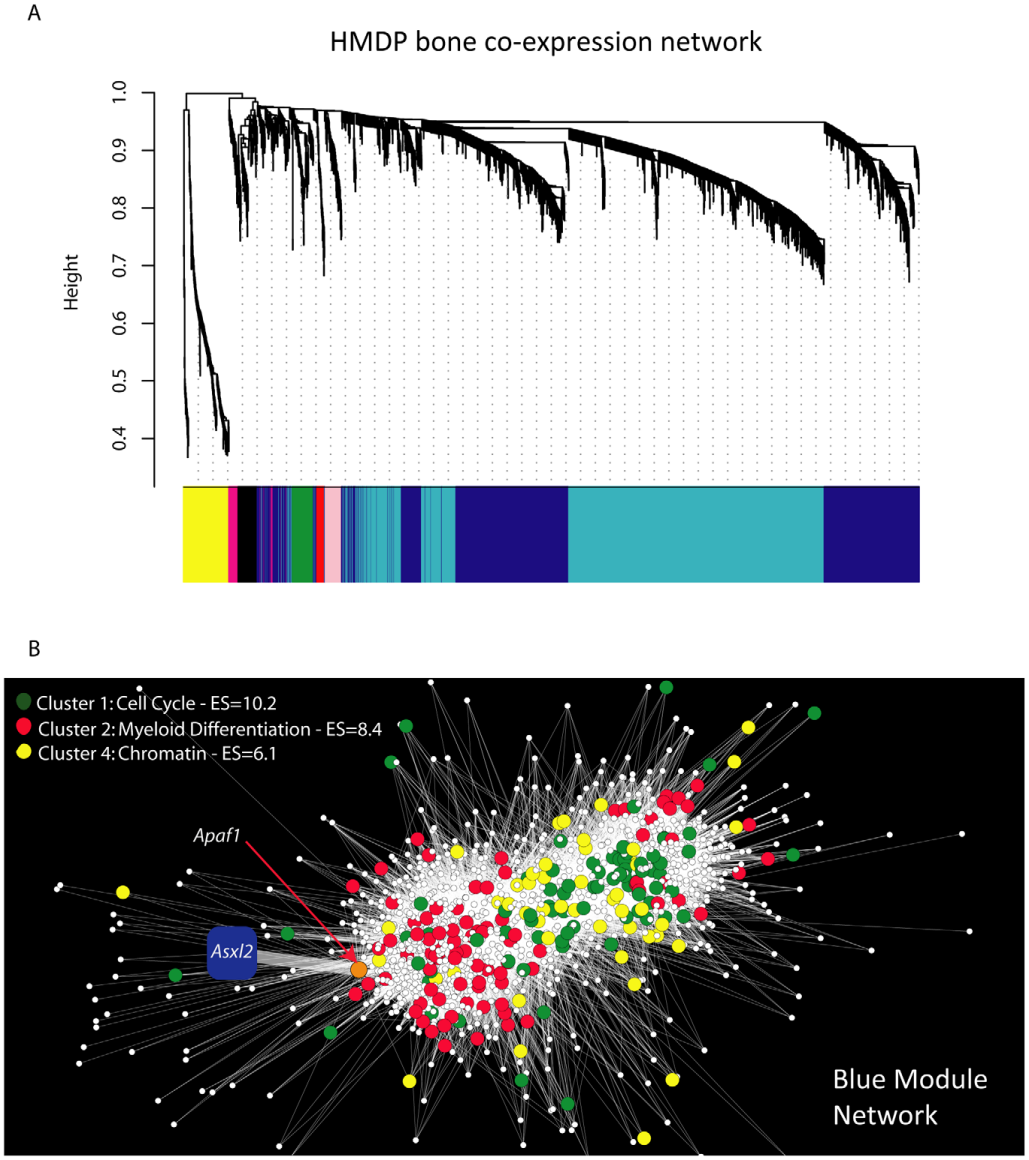
Gene co-expression network analysis reveals that *Asxl2* is connected to genes involved in myeloid cell differentiation. (A) Gene co-expression network of the 3600 most variable and connected genes in the bone transcriptome. The network was created using Weighted Gene Co-expression Network Analysis (WGCNA). Genes were plotted based on a dissimilarity metric (1-TOM). The low-hanging gene “branches” (each gene is represented by a single line) are groups of genes that have highly similar TOMs (i.e. low dissimilarity). Based on the structure of each branch genes are placed into a “module” of a particular color. Each branch's color is labeled at the bottom of the panel. *Asxl2* was a member of the blue module. (B) Node and edge depiction of the 34,690 edges (i.e. connections between nodes or genes) with the highest TOM in the blue module network. The blue module is enriched for genes involved in the cell cycle (green nodes), myeloid cell differentiation (red nodes) and chromatin (yellow nodes). The blue square corresponds to *Asxl2*. *Asxl2* is connected to one other gene, *Apaf1*; the orange node. *Apaf1*, in turn, is connected to 149 genes in the cluster of genes on the left side of the network. This group is enriched (P = 2.0×10^−3^) for genes involved in myeloid cell differentiation (red nodes).

We next used systems genetics to begin to identify a potential function for *Asxl2* in bone. For this analysis we utilized the cortical bone gene expression data from the HMDP strains. An analysis of *Asxl2* expression revealed that while its expression was not under regulation by detectable local (described above) or distant eQTL (data not shown), *Asxl2* was expressed in cortical bone (in the top 10% of probes based on average expression) and most importantly, its expression differed by 1.5-fold between the lowest and highest expressing HMDP strains (Figure S1). We reasoned that its high level of expression and variation in expression among strains would allow us to identify biologically meaningful co-expression relationships between *Asxl2* and genes sharing similar functions, even though a difference in its expression does not underlie the Chr. 12 association. To identify the genes that were co-expressed with *Asxl2* in bone, genes were grouped into “co-expression modules” using Weighted Gene Co-expression Network Analysis (WGCNA) [20]. We used WGCNA to generate a bone co-expression network comprised of the 3600 most variable and highly connected genes (see Methods). The 3600 genes were subsequently partitioned into eight gene modules (Figure 5A). Of the eight, we focused our attention on the blue module that contained *Asxl2* along with 1334 other genes (full list is provided in Table S4). The DAVID knowledge base (http://david.abcc.ncifcrf.gov/) was used to determine if the blue module was enriched for specific gene ontology (GO) categories. We were most interested in identifying enrichments in specific gene functions; thus, we restricted the analysis to the GO biological process and molecular function categories and excluded the cellular component category. DAVID's functional annotation clustering tool was used to identify 14 significant (enrichment score (ES) >3.0; see Methods) gene clusters containing highly related GO terms (Table S5). The top four clusters contained genes involved in: 1) the cell cycle/chromosome/DNA replication/cell division, 2) hematopoiesis/myeloid cell differentiation, 3) ATP binding and 4) chromosome organization/chromatin organization (Table 4). *Asxl2* is thought to regulate the function of Polycomb (PcG) and Trithorax (trxG) protein complexes, which are involved in the establishment and maintenance of chromatin [31]. Its membership in a module enriched for genes involved in the GO terms “chromosome” (Bonferroni corrected enrichment P = 3.2×10^−7^) and “chromatin organization” (Bonferroni corrected enrichment P = 9.2×10^−3^) is consistent with its known function and suggests that this module is comprised of biologically meaningful co-expression relationships.

**Table 4:** Top four functional annotation clusters for the blue module.

Annotation Cluster 1: Enrichment Score = 10.1^a^	Count^b^	%^c^	FE^d^	P-value^e^
GO:0007049∼cell cycle	105	8.2	2.6	6.8×10^−16^
GO:0051301∼cell division	55	4.3	2.9	4.0×10^−9^
GO:0000278∼mitotic cell cycle	48	3.7	2.9	1.1×10^−7^
GO:0022402∼cell cycle process	63	4.9	2.4	5.9×10^−7^
GO:0022403∼cell cycle phase	55	4.3	2.5	1.9×10^−6^
GO:0000087∼M phase of mitotic cell cycle	39	3.0	3.0	5.0×10^−6^
GO:0048285∼organelle fission	39	3.0	2.9	7.8×10^−6^
GO:0007067∼mitosis	38	3.0	3.0	9.6×10^−6^
GO:0000280∼nuclear division	38	3.0	3.0	9.6×10^−6^
GO:0000279∼M phase	47	3.7	2.5	5.2×10^−5^
**Annotation Cluster 2: Enrichment Score = 8.4**				
GO:0030218∼erythrocyte differentiation	19	1.5	5.9	2.0×10^−6^
GO:0002376∼immune system process	100	7.8	1.9	2.2×10^−6^
GO:0030097∼hemopoiesis	46	3.6	2.7	3.2×10^−6^
GO:0034101∼erythrocyte homeostasis	19	1.5	5.5	6.3×10^−6^
GO:0002520∼immune system development	50	3.9	2.5	8.6×10^−6^
GO:0048534∼hemopoietic or lymphoid organ development	48	3.7	2.5	1.4×10^−5^
GO:0030099∼myeloid cell differentiation	25	1.9	3.9	3.8×10^−5^
GO:0048872∼homeostasis of number of cells	25	1.9	3.3	1.0×10^−3^
**Annotation Cluster 3: Enrichment Score = 8.0**				
GO:0032553∼ribonucleotide binding	182	14.2	1.6	6.2×10^−8^
GO:0032555∼purine ribonucleotide binding	182	14.2	1.6	6.2×10^−8^
GO:0017076∼purine nucleotide binding	187	14.5	1.6	1.0×10^−7^
GO:0000166∼nucleotide binding	209	16.3	1.5	2.8×10^−7^
GO:0032559∼adenyl ribonucleotide binding	148	11.5	1.6	6.0×10^−6^
GO:0005524∼ATP binding	146	11.4	1.6	8.8×10^−6^
GO:0030554∼adenyl nucleotide binding	153	11.9	1.6	1.0×10^−5^
GO:0001883∼purine nucleoside binding	153	11.9	1.6	1.8×10^−5^
GO:0001882∼nucleoside binding	153	11.9	1.5	2.7×10^−5^
GO:0016301∼kinase activity	90	7.0	1.7	3.7×10^−4^
GO:0016772∼transferase activity, transferring phosphorus-containing groups	98	7.6	1.6	2.3×10^−3^
GO:0016773∼phosphotransferase activity, alcohol group as acceptor	72	5.6	1.6	4.5×10^−2^
**Annotation Cluster 4: Enrichment Score = 6.1**				
GO:0051276∼chromosome organization	65	5.1	2.4	2.5×10^−7^
GO:0006325∼chromatin organization	45	3.5	2.1	8.1×10^−3^
GO:0016568∼chromatin modification	29	2.3	1.8	1.0

**^a^**Geometric mean (in −log scale) of the nominal p-values (not shown) for each GO category in a cluster.

**^b^**Number of GO category genes in the blue module.

**^c^**Percentage of GO category genes in the blue module.

**^d^**Fold Enrichment of genes in the blue modules compared to a background list.

**^e^**Bonferroni corrected P-value for the enrichment of each GO category in the blue module.

We next characterized the nature of the genes most closely connected to *Asxl2* in the blue module. For this purpose, a network view was generated for the 34,690 strongest connections among 1256 (94%) of the 1335 blue module genes (Figure 5B). In this view of the blue module, *Asxl2* was connected to a single node, the apoptotic peptidase-activating factor 1 (*Apaf1*) gene. *Apaf1*, in turn, was connected to 149 genes; all located within the cluster of genes on the left side of the network depiction Figure 5B. To examine the gene composition of the cluster connected to *Asxl2*, genes were colored based on their membership in three of the four top enrichments described above, including the “cell cycle”, “myeloid cell differentiation” and “chromatin organization” (Table 4). We excluded the “ATP-binding category” since genes in this category are involved in a wide-range of biological processes. The blue module as a whole was enriched for all three categories (Table 4); however, the cluster of genes most closely connected to *Asxl2* contained most (75%; enrichment P = 2.0×10^−3^) of the genes involved in myeloid cell differentiation (red nodes in Figure 5B) and very few cell cycle (green nodes) or chromatin organization (yellow nodes) genes. These data indicate that in our bone network, *Asxl2* is most closely connected with genes involved in myeloid cell differentiation and may play a role in this process.

### *Asxl2* is a regulator of osteoclastogenesis

**Figure 6:**
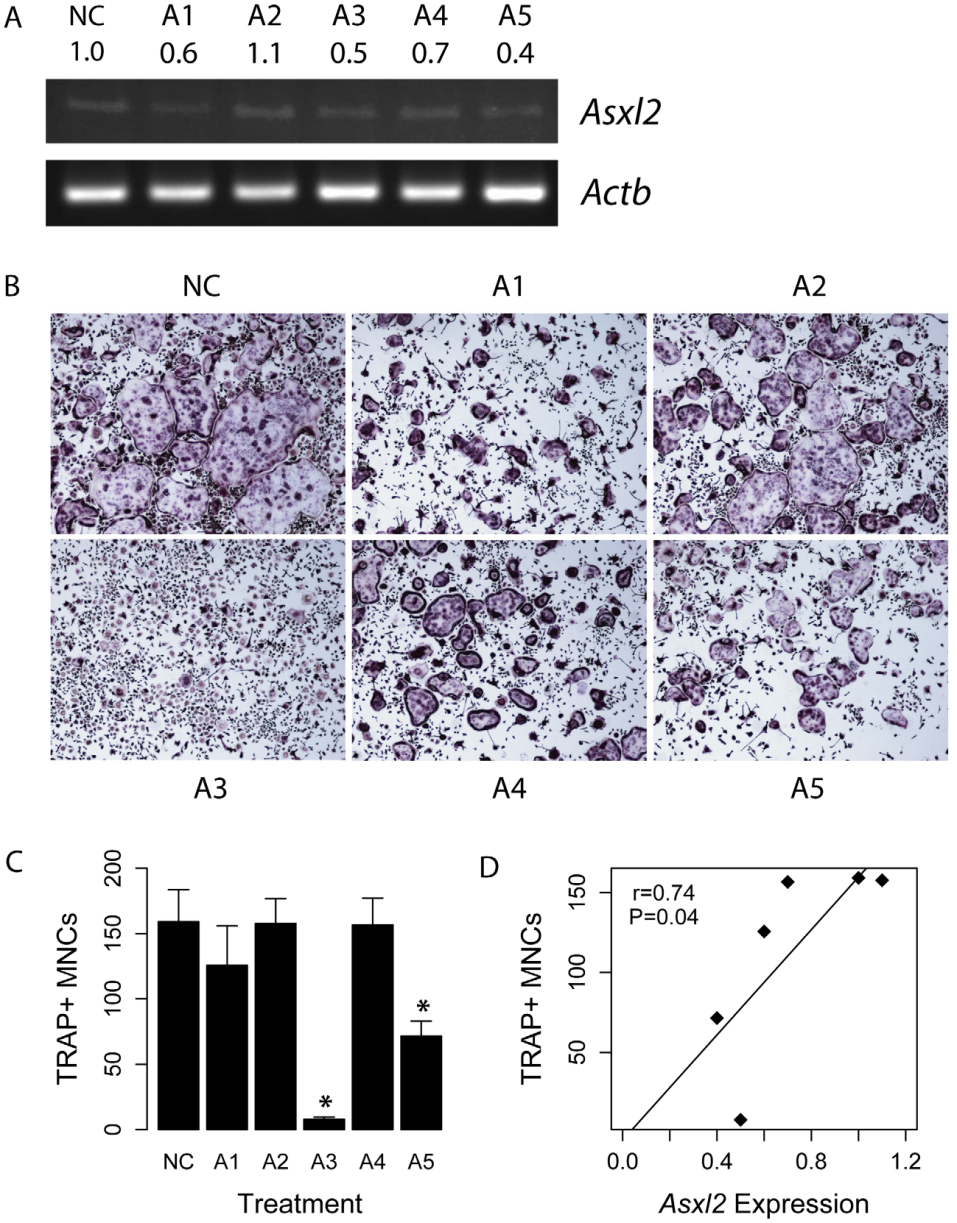
*Asxl2* is a regulator of osteoclastogenesis. (A) RT-PCR analysis of *Asxl2* expression in BMMs infected with a control (NC) or five distinct (A1–A5) lentiviral shRNA constructs targeting *Asxl2*. Numbers below construct names represent densitometrically determined relative *Asxl2* expression ratios normalized for *Actb*. (B) TRAP staining of BMMs transduced with NC or one of the five (A1–A5) *Asxl2* lentiviral shRNA constructs five days after induction of osteoclast differention with M-CSF and RANKL. (C) Quantification of TRAP+ MNCs for cells shown in (B) (N = 6/treatment). (D) Relative expression of *Asxl2* is positively correlated (r = 0.74; P = 0.04) with the number of TRAP+ MNCs across the five infections. Data shown in (C) represents mean±SEM. *P<0.05.

The GO category “myeloid cell differentiation” is comprised of genes that are involved in the general process of myeloid precursors acquiring characteristics of downstream cell lineages. With respect to bone cells, osteoclasts are bone-resorbing cells of myeloid origin; thus, we hypothesized that *Asxl2* played a role in the differentiation of pre-osteoclasts. Additionally, many of the blue module genes in this category have been implicated in osteoclastogenesis, such as *Inpp5d* (aka SHIP) [32], *Smad5* [33], *Id2* [34], *Plcg2* [35], *Twsg1* [36], among others. To test the hypothesis that *Asxl2* was involved in osteoclastogenesis, we infected bone marrow macrophages (BMMs) with lentiviral constructs expressing short-hairpin RNAs (shRNA) targeting *Asxl2*. BMMs were infected with a vector only control (NC) or one of five lentiviral constructs (A1–A5) expressing distinct shRNAs targeting *Asxl2*. After five days of culture, *Asxl2* expression was particularly lower in cells infected with the A3 and A5 lentiviral constructs (Figure 6A). Osteoclastogenesis was induced in infected BMMs by culturing in the presence of M-CSF and RANKL. After five days of culture the number of TRAP+ (tartrate-resistant acid phosphatase, a marker of osteoclasts) multinuclear cells (MNCs) was significantly (P<0.05) reduced in cultures infected with lentiviral constructs A3 and A5 compared to NC treated cells (Figure 6B and 6C). We observed a strong positive correlation (r = 0.74, P = 0.04) between the relative expression of *Asxl2* and TRAP+ MNCs across the six treatments (Figure 6D). These data are consistent with our network inference and confirm that *Asxl2* is a regulator of osteoclastogenesis and strengthen the hypothesis that *Asxl2* is a regulator of BMD.

## Discussion

The mouse has numerous advantages for the genetic analysis of BMD; however, historically mapping approaches in the mouse have been plagued by the lack of resolution. Additionally, GWA approaches provide no information on the function of associated genes. We have addressed both limitations through the use of high-resolution GWA in the HMDP to identify associations confined to narrow genomic intervals and gene co-expression analysis of bone microarray data to provide insight on gene function. This novel analytical paradigm resulted in the discovery of *Asxl2* as a regulator of BMD and osteoclastogenesis. This study identified a new gene and possibly an entire network of genes that play an important role in BMD and osteoclast function.

Polycomb (PcG) and trithorax (trxG) are highly conserved protein complexes that are involved in the repression and activation of gene expression, respectively, through the establishment and maintenance of chromatin modifications at specific target genes [37]. With respect to bone cells, PcG and trxG have been implicated in cell proliferation [38], myeloid precursor maturation [39] and osteoblast differentiation [40]. In *Drosophila*, *Additional sex combs* (*Asx*) belongs to a group of proteins known as the Enhancers of trxG and PcG (ETP) [41]. Although its specific mechanism is unknown, *Asx* is thought to promote both PcG-mediated silencing and trxG-mediated activation of gene expression. In humans and mice there are three *Asx* homologues, *Asx-like 1*, *2* and *3* [42], [43], [44]. Mutations in *ASXL1* result in myleoproliferative neoplasms [45] and little is known regarding the function of *ASXL3*. Recently described *Asxl2* ^−/−^ knockout mice display a global reduction in the PcG-associated histone modification trimethylation of histone H3 lysine 27. This is consistent with a conserved function as an ETP in mammals [31]. Additionally, *Asxl2^−/−^* mice develop anterior and posterior transformations of the axial skeleton [31], which further supports our observation that *Asxl2* is involved in bone development.

An important open question is whether *Asxl2* impacts bone development through its expression in other cell-types, such as osteoblasts. If *Asxl2* functioned exclusively in osteoclasts, we would expect based on the *in vitro* osteoclastogenesis data, that loss of *Asxl2* function would impair osteoclast function and bone resorption *in vivo*, resulting in increased BMD. In contrast, we observed a decrease in BMD in *Asxl2^−/−^* mice. It has been shown in a number of mouse models that loss of specific genes can lead to an impairment of both osteoblast and osteoclast function. This results in a condition referred to as low-turnover osteopenia in which bone formation by osteoblasts and resorption by osteoclasts are impaired with a net loss of bone. As examples, osteoblasts and osteoclasts from mice deficient in *klotho* [46], *JunB* [47] and *Akt1* [48] have impaired *in vitro* differentiation. These mice also have reduced BMD due to low-turnover osteopenia. In addition, Synaptotagmin VII (*Syt11*) has been shown to alter protein secretion in osteoblasts and osteoclasts, resulting in decreased bone mass in *Syt^−/−^* mice [49]. In data not presented we observed (using publically available data from the BioGPS browser; http://biogps.gnf.org, probes 1460597_at and 1439063_at) high and ubiquitous expression of *Asxl2* in 96 different mouse tissues and cell-lines, including primary osteoclasts and osteoblasts. In addition, while the blue module was highly enriched for genes involved in myeloid differentiation there were also a number of genes such as *Bmp4*, *Chrd*, *Hdac5* and *Igf2* that play a role in osteoblast differentiation (Table S3). These data suggest that the decreases in BMD seen in *Asxl2^−/−^* mice may be due to deficiencies in both osteoclast and osteoblast function. Further work is needed to clarify the precise role of *Asxl2* in bone.

We have previously characterized the HMDP as a novel population for GWA in the mouse [15]. This study extends our original observations by demonstrating the feasibility of identifying associations affecting additional complex phenotypes. In contrast to more traditional mouse linkage mapping strategies, we used association in the HMDP to identify four associations containing a relatively small number of genes. Based on prior work, the boundaries of the associations were defined as the 2.6 Mb region surrounding the most significant SNP. We expect that these intervals are conservative and would likely be smaller if based on region specific LD patterns, as shown for Chr. 12 (Figure 3). However, defining the associations in this way allowed us to be confident that the regions contained the causal gene(s). This study also highlights the other key advantage of the HMDP; the ability to collect and molecularly profile tissues, such as bone, that are difficult or impossible to collect from a single mouse or in human populations. In the future, the accumulation of many bone-related clinical and molecular phenotypes in the HMDP will enable the large-scale systems-level analyses that should provide significant insight on bone physiology and genetics. Additionally, the HMDP will provide the opportunity to address the genetic basis of extremely important phenotypes, such as bone loss, nanostrucutal properties of bone and bone cell aging that are difficult to address in humans. Moreover, as we have used systems genetic to gain functional insight for a gene identified by mouse GWA, it is also possible that the HMDP could be used to dissect the function of genes identified in human GWA studies.

However, the HMDP is not without limitations. First, the statistical power to detect effects of subtle variants is modest. We have previously estimated that for highly heritable phenotypes, such as BMD, the power to detect variants explaining 10% of the trait variance is 50% [15]. The power drops precipitously for variants explaining less than 10% of the variance. Thus, this version of the HMDP (with ∼100 strains) is unable to identify the many more variants with subtle effects that are undoubtedly affecting BMD in this population. This problem will be less of an issue for future HMDP panels containing a larger number of strains. Additionally, one of the side effects of the breeding history of inbred mouse strains is the presence of LD between markers on separate chromosomes (i.e. non-syntenic LD). It is thought that this is due to selection for allelic combinations that confer increased fitness during the inbreeding process [50]. False-positive associations can arise if a region associated with a phenotype is in LD with other regions of the genome. Although this is always a potential pitfall when using the HMDP, it is easily identifiable and we did not observe LD (r^2^<0.4) between any of the four BMD associations (data not shown).

Most GWA studies stop at gene discovery. However, without physiologically relevant functional information it is often difficult to be confident that the true causal gene has been identified and to begin unraveling the mechanistic underpinnings of significant associations. Recently, some GWA studies have begun to incorporate gene expression information to determine if a significant variant regulates expression. A positive finding in such an analysis suggests that the genotype dependent differential gene expression is the basis of the association. This approach has recently been used to discover that variants in the promoter of the serine racemase (*SRR*) gene regulate its expression and BMD [51]. We have taken that application one step further and developed a bone transcriptional network to aid in the functional annotation of genes of unknown function. Using this network, we were able to determine that *Asxl2* was closely connected to genes with links to osteoclast differentiation. This simple connection allowed us to test the hypothesis that *Asxl2* was involved in a bone specific function. Another important point is that, as we demonstrated for *Asxl2*, a gene's expression does not have to be under genetic regulation for this approach to work.

In conclusion, we have used mouse GWA and gene co-expression network analysis to identify *Asxl2* as a novel regulator of BMD and osteoclastogenesis. Our analysis has revealed a new gene and pathway that play an important role in bone development. Additionally, this study demonstrates the feasibility of using the HMDP for the dissection of complex genetic traits.

## Materials and Methods

### Ethics statement

The animal protocol for the HMDP mice was approved by the Institutional Care and Use Committee (IACUC) at the University of California, Los Angeles. The animal protocol for the *Asxl2^−/−^* mice was approved by the Animal Care Committee (ACC) at the University of Illinois at Chicago.

### The Hybrid Mouse Diversity Panel

Approximately nine male mice for each HMDP strain (Table S1) were purchased from the Jackson Labs (Bar Harbor, ME). Mice were between 6 and 10 weeks of age and to ensure adequate acclimatization to a common environment the mice were aged until 16 weeks before sacrifice. All mice were maintained on a chow diet (Ralston-Purina Co, St. Louis, Mo) until sacrifice.

### Genotyping

Inbred strains were previously genotyped by the Broad Institute (available via www.mousehapmap.org). Genotypes of recombinant inbred strains were imputed as previously described [15]. Of the 140,000 SNPs available at the Broad Institute, 108,064 were informative with an allele frequency ≥5% and less than 20% missing data, and were used for the association analysis.

### BMD determination

All carcasses were stored at −20°C after sacrifice and then thawed overnight at 4°C prior to BMD scans. The entire thawed carcass was scanned. BMD scans were performed using a Lunar PIXImus II Densitometer (GE Healthcare, Piscataway, NJ). The PIXImus II was calibrated daily using a phantom of known BMD. BMD was calculated for the entire carcass minus the skull, the lumbar spine and the left femur.

### RNA and microarray processing

At sacrifice the diaphysis of the right femur was excised and cleaned free of soft tissue. Bone marrow was removed by flushing with PBS using a 22-guage needle and 3 ml syringe. The bone was then flash frozen in LN_2_ and stored at −80C. Total RNA was isolated using the Trizol Plus RNA Purification Kit (Invitrogen, Carlsbad, CA) following homogenization of the whole bone sample. RNA integrity was confirmed using the Agilent 2100 Bioanalyzer (Agilent, Palo Alto, CA). Microarray expression profiles were generated (N = 1–3 per strain) using the Illumina MouseWG-6 v1.1 BeadChips (Illumina, San Diego, CA) by the Southern Genotyping Consortium at UCLA. Biotin-labeled cRNA was synthesized by the total prep RNA amplification kit from Ambion (Austin, TX). cRNA was quantified and normalized to 77 ng/µl, and then 850 ng was hybridized to Beadchips.

### Microarray data processing

The expression values were transformed using the Variance Stabilizing Transformation (VST) [52], and normalized with the Robust Spline Normalization (RSN) algorithm using the LumiR R package [53]. After normalization, the ComBat software was used to adjust for batch effects using an empirical Bayes methods [54]. Microarray data has been submitted the the NCBI Gene Expression Omnibus (GEO) database (GSE27483).

### EMMA

Population structure is a major confounding for genome-wide association analyses in the HMDP. This is due to the fact that many phenotypes correlate with the phylogeny of HMDP strains (i.e. genetically similar strains have similar phenotypes) and any SNP that correlates with these strain relationships will be falsely associated with the phenotype. The Efficient Mixed-Model Association (EMMA) algorithm has been shown to effectively reduce this confounding [23]. We applied the following linear mixed model to perform association mapping between BMD or a gene's expression and a marker under the confounding effect from population structure [23], [55], [56]; 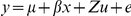, where 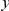 is a phenotypes of each mouse, 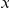 is their genotype, 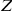 is an incidence matrix mapping each mouse to corresponding strain, 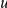 is a random effect accounting for population structure effect with 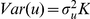, and 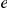 is the uncorrelated random effect with 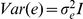. The Efficient Mixed Model Association (EMMA) [23] is used for efficient and reliable estimation of restricted maximum likelihood (REML) parameters and hypothesis testing under the linear mixed model. After estimating REML parameters, a standard F test is used to test the statistical significance of the marker-phenotype association.

### Candidate gene characterization

To characterize the genes located in each BMD association, we downloaded all RefSeq genes in the four regions from the USCS genome browser (http://genome.ucsc.edu/cgi-bin/hgGateway) using the NCBI Build37 genome assembly. From the Illumina MouseWG-6 microarray we identified all probes corresponding to the 163 RefSeq genes. Probes were excluded if they overlapped with SNPs (dbSNP 128) to avoid the hybridization artifacts that can arise due [57], [58]. EMMA was used to calculate association P-values for all probes corresponding to the 163 RefSeq genes. Only SNPs mapping to each associated region were used in this analysis. Known non-synonymous SNPs within each region were downloaded from the Mouse Phenome Database (http://phenome.jax.org/) using a set of over 7 million genotyped and imputed SNPs. We only selected SNPs that were variant in at least one of the classical inbred strains represented in the HMDP. Prediction of the functional effect of these SNPs was performed using the PolyPhen tool (http://genetics.bwh.harvard.edu/pph/). R^2^ was calculated for each non-synonymous SNP and the peak BMD SNP within the 30 classical inbred strains in the HMDP.

### Characterizing BMD in *Asxl2^−/−^* mice

The generation and initial characterization of *Asxl2^−/−^* gene trap mice has been previously described [31]. These mice harbor a gene trap cassette downstream of exon 1. Homozygotes for the gene trap allele show little (∼3%) *Asxl2* expression whereas heterozygotes expressed *Asxl2* at ∼50% of wild type levels. BMD in littermate male and female mice (2–4 months of age) of varying *Asxl2* genotype was measured as described above. Age and weight at sacrifice were recorded. To assess the effects of *Asxl2* deficiency on BMD independent of age and weight we generated BMD residuals using a simple linear regression. A Student's t-test was used to test the significance of the differences in BMD residuals in the different genotypes.

### Weighted Gene Co-Expression Network Analysis

Network analysis was performed using the WGCNA R package [59]. An extensive overview of WGCNA, including numerous tutorials, can be found at http://www.genetics.ucla.edu/labs/horvath/CoexpressionNetwork/. To begin, we filtered the array data to remove lowly and non-expressed genes by selecting probes based on a detection P-value of <0.05 in 95% of the samples. Next, we selected the 8000 most varying genes based on variance across the 95 samples and then selected the most connected (based on k.total described below) 3600 genes for network analysis. Our group and others have used this number of genes previously (as examples [19], [60]), mainly because most other genes have very low k.total values. If multiple probes existed for a given gene only the most connected probe per gene was included in the list of 3600. To generate a co-expression network for the selected probes, we first calculated Pearson correlation coefficients for all gene-gene comparisons across the 95 microarray samples. The matrix of correlations was then converted to an adjacency matrix of connection strengths. The adjacencies were defined as 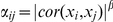 where 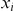 and 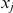 are the 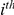 and 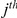 gene expression traits. The power 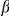 was selected using the scale-free topology criterion previously outlined by Zhang and Horvath [18]. Network connectivity (k.total) of the 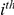 gene was calculated as the sum of the connection strengths with all other network genes, 
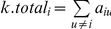
. This summation performed over all genes in a particular module was defined as the intramodular connectivity (k.in). Modules were defined as sets of genes with high topological overlap [18]. The topological overlap measure (TOM) between the 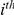 and 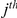 gene expression traits was taken as 
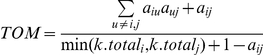
, where 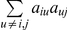 denotes the number of nodes to which both 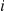 and 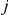 are connected, and 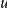 indexes the nodes of the network. A TOM-based dissimilarity measure 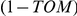 was used for hierarchical clustering. Gene modules corresponded to the branches of the resulting dendrogram and were precisely defined using the “Dynamic Hybrid” branch cutting algorithm [61]. Highly similar modules were identified by clustering and merged together. In order to distinguish modules each was assigned a unique color.

### Osteoclast culture and lentiviral transduction

Whole bone marrow was extracted from femora of mice with α-MEM and cultured overnight in α-MEM (Sigma-Aldrich, St Louis, MO) containing 10% heat-inactivated FBS, 100 IU/ml penicillin, and 100 µg/ml streptomycin (α10 medium). The nonadherent cells were collected by centrifugation and re-plated in a new 10-cm petri dish in α10 medium. To generate osteoclasts, 100 ng/ml RANKL and 1/100 vol CMG 14-12 culture supernatant were added to α10 medium for 4–5 days. Osteoclasts were stained for TRAP as described by the manufacturer's instructions (Sigma-Aldrich). The SHCLNG-NM_172421 MISSION lentiviral shRNA (Sigma-Aldrich) clone set (containing five separate lentiviral shRNA clones each expressing a distinct *Asxl2* targeting sequence) was used to transduce BMMs according to manufacturer's specifications. The MISSION pLKO.1-puro control vector was used as a negative control.

### Semi-quantitative RT-PCR

Total RNA was isolated from cultures using the RNeasy Mini Kit (Qiagen). Purified RNA was DNase treated using the DNA-free kit (Ambion). The High Capacity cDNA Reverse Transcription Kit (Applied Biosystems) was used to synthesize cDNA in a volume of 20 µl. The reaction mixture was adjusted to 200 µl with dH_2_O and 5 µl of the dilute cDNA was used for PCR. PCR was performed for 22 cycles for *Asxl2* and *Actb* using the following primers (Asxl2-F, 5′-ACCCACCATTCCAGCAAGTA-3′, Asxl2-R, 5′-TGGCTGCTTTGACAGTCTTG-3′, Actb-F, 5′-CCAACCGTGAAAAGATGACC-3′, Actb-R, 5′-ACCAGAGGCATACAGGGACA-3′). PCR products were separated on a 1.5% agarose gel containing 0.5 mg/ml ethidium bromide. Band densitometry was performed using the Image J software (NIH). Normalized *Asxl2* expression levels were determined by subtracting lane specific backgrounds for each sample and dividing by *Actb* intensities.

## Supporting Information

Figure S1 The expression of *Asxl2* across HMDP strain is highly variable. *Asxl2* expression values from cortical bone (femoral diaphysis with marrow removed) microarray data from 95 HMDP strains. *Asxl2* was highly expressed in bone and its expression varies by 1.5-fold (log2 difference of 0.6) in the lowest and highest expressing strains.
(PDF)

Table S1 List of individual BMD data points for all HMDP mice used in the analysis.
(XLSX)

Table S2 List of eQTL mapping results for genes located within the four BMD GWA associations. The local eQTL P-value column contains the most significant association between the all SNPs located within eac association and the expression of each probe from the region. The overlapping SNP column contains the number of SNPs that overlap each probe.
(XLSX)

Table S3 List of all non-synonymous SNPs within the 163 GWA candidate genes. PolyPhen assignments; B, Benign; Poss D, Possibly Damaging and Prob D, Probably Damaging. R^2^ was calculated for each NS SNP and the peak marker within its respective region.
(XLSX)

Table S4 List of metrics for all blue module genes. R with *Asxl2* equals the correlation between the expression of each network gene and *Asxl2*.
(XLSX)

Table S5 List of DAVID functional clusters with enrichment scores >3.0 for the blue module.
(XLSX)

## References

[1] (2001). Osteoporosis prevention, diagnosis, and therapy.. JAMA.

[2] Johnell O, Kanis JA, Oden A, Johansson H, De Laet C (2005). Predictive value of BMD for hip and other fractures.. J Bone Miner Res.

[3] Ralston SH (2007). Genetics of osteoporosis.. Proc Nutr Soc.

[4] Xiong Q, Jiao Y, Hasty KA, Canale ST, Stuart JM (2009). Quantitative trait loci, genes, and polymorphisms that regulate bone mineral density in mouse.. Genomics.

[5] Flint J, Valdar W, Shifman S, Mott R (2005). Strategies for mapping and cloning quantitative trait genes in rodents.. Nat Rev Genet.

[6] Klein RF, Allard J, Avnur Z, Nikolcheva T, Rotstein D (2004). Regulation of bone mass in mice by the lipoxygenase gene Alox15.. Science.

[7] Nakanishi R, Shimizu M, Mori M, Akiyama H, Okudaira S (2006). Secreted frizzled-related protein 4 is a negative regulator of peak BMD in SAMP6 mice.. J Bone Miner Res.

[8] Edderkaoui B, Baylink DJ, Beamer WG, Wergedal JE, Porte R (2007). Identification of mouse Duffy antigen receptor for chemokines (Darc) as a BMD QTL gene.. Genome Res.

[9] Ackert-Bicknell CL, Karasik D, Li Q, Smith RV, Hsu YH (2010). Mouse BMD quantitative trait loci show improved concordance with human genome-wide association loci when recalculated on a new, common mouse genetic map.. J Bone Miner Res.

[10] Liu P, Wang Y, Vikis H, Maciag A, Wang D (2006). Candidate lung tumor susceptibility genes identified through whole-genome association analyses in inbred mice.. Nat Genet.

[11] Cheng R, Lim JE, Samocha KE, Sokoloff G, Abney M (2010). Genome-wide Association Studies and the Problem of Relatedness Among Advanced Intercross Lines and Other Highly Recombinant Populations.. Genetics.

[12] Valdar W, Solberg LC, Gauguier D, Burnett S, Klenerman P (2006). Genome-wide genetic association of complex traits in heterogeneous stock mice.. Nat Genet.

[13] Farber CR, van Nas A, Ghazalpour A, Aten JE, Doss S (2009). An integrative genetics approach to identify candidate genes regulating BMD: combining linkage, gene expression, and association.. J Bone Miner Res.

[14] Ghazalpour A, Doss S, Kang H, Farber C, Wen PZ (2008). High-resolution mapping of gene expression using association in an outbred mouse stock.. PLoS Genet.

[15] Bennett BJ, Farber CR, Orozco L, Kang HM, Ghazalpour A (2010). A high-resolution association mapping panel for the dissection of complex traits in mice.. Genome Res.

[16] Schadt EE (2009). Molecular networks as sensors and drivers of common human diseases.. Nature.

[17] Farber CR, Lusis AJ (2009). Future of osteoporosis genetics: enhancing genome-wide association studies.. J Bone Miner Res.

[18] Zhang B, Horvath S (2005). A general framework for weighted gene co-expression network analysis.. Stat Appl Genet Mol Biol.

[19] Ghazalpour A, Doss S, Zhang B, Wang S, Plaisier C (2006). Integrating genetic and network analysis to characterize genes related to mouse weight.. PLoS Genet.

[20] Horvath S, Zhang B, Carlson M, Lu KV, Zhu S (2006). Analysis of oncogenic signaling networks in glioblastoma identifies ASPM as a molecular target.. Proc Natl Acad Sci U S A.

[21] Oldham MC, Konopka G, Iwamoto K, Langfelder P, Kato T (2008). Functional organization of the transcriptome in human brain.. Nat Neurosci.

[22] Wolfe CJ, Kohane IS, Butte AJ (2005). Systematic survey reveals general applicability of “guilt-by-association” within gene coexpression networks.. BMC Bioinformatics.

[23] Kang HM, Zaitlen NA, Wade CM, Kirby A, Heckerman D (2008). Efficient control of population structure in model organism association mapping.. Genetics.

[24] Bennett BJ, Farber CR, Orozco L, Kang HM, Ghazalpour A A high-resolution association mapping panel for the dissection of complex traits in mice.. Genome Res.

[25] Ramensky V, Bork P, Sunyaev S (2002). Human non-synonymous SNPs: server and survey.. Nucleic Acids Res.

[26] Sunyaev S, Ramensky V, Koch I, Lathe W, Kondrashov AS (2001). Prediction of deleterious human alleles.. Hum Mol Genet.

[27] Sabatakos G, Sims NA, Chen J, Aoki K, Kelz MB (2000). Overexpression of DeltaFosB transcription factor(s) increases bone formation and inhibits adipogenesis.. Nat Med.

[28] Soysa NS, Alles N, Weih D, Lovas A, Mian AH (2009). The Pivotal Role of the Alternative NF-kappaB Pathway in Maintenance of Basal Bone Homeostasis and Osteoclastogenesis.. J Bone Miner Res.

[29] Schilling AF, Schinke T, Munch C, Gebauer M, Niemeier A (2005). Increased bone formation in mice lacking apolipoprotein E.. J Bone Miner Res.

[30] Ducy P, Zhang R, Geoffroy V, Ridall AL, Karsenty G (1997). Osf2/Cbfa1: a transcriptional activator of osteoblast differentiation.. Cell.

[31] Baskind HA, Na L, Ma Q, Patel MP, Geenen DL (2009). Functional conservation of asxl2, a murine homolog for the Drosophila enhancer of trithorax and polycomb group gene asx.. PLoS ONE.

[32] Zhou P, Kitaura H, Teitelbaum SL, Krystal G, Ross FP (2006). SHIP1 negatively regulates proliferation of osteoclast precursors via Akt-dependent alterations in D-type cyclins and p27.. J Immunol.

[33] Kaneko H, Arakawa T, Mano H, Kaneda T, Ogasawara A (2000). Direct stimulation of osteoclastic bone resorption by bone morphogenetic protein (BMP)-2 and expression of BMP receptors in mature osteoclasts.. Bone.

[34] Lee J, Kim K, Kim JH, Jin HM, Choi HK (2006). Id helix-loop-helix proteins negatively regulate TRANCE-mediated osteoclast differentiation.. Blood.

[35] Mao D, Epple H, Uthgenannt B, Novack DV, Faccio R (2006). PLCgamma2 regulates osteoclastogenesis via its interaction with ITAM proteins and GAB2.. J Clin Invest.

[36] Sotillo Rodriguez JE, Mansky KC, Jensen ED, Carlson AE, Schwarz T (2009). Enhanced osteoclastogenesis causes osteopenia in twisted gastrulation-deficient mice through increased BMP signaling.. J Bone Miner Res.

[37] Schuettengruber B, Chourrout D, Vervoort M, Leblanc B, Cavalli G (2007). Genome regulation by polycomb and trithorax proteins.. Cell.

[38] Martinez AM, Cavalli G (2006). The role of polycomb group proteins in cell cycle regulation during development.. Cell Cycle.

[39] Arai S, Miyazaki T (2005). Impaired maturation of myeloid progenitors in mice lacking novel Polycomb group protein MBT-1.. Embo J.

[40] Zhang HW, Ding J, Jin JL, Guo J, Liu JN (2010). Defects in mesenchymal stem cell self-renewal and cell fate determination lead to an osteopenic phenotype in Bmi-1 null mice.. J Bone Miner Res.

[41] Gildea JJ, Lopez R, Shearn A (2000). A screen for new trithorax group genes identified little imaginal discs, the Drosophila melanogaster homologue of human retinoblastoma binding protein 2.. Genetics.

[42] Fisher CL, Randazzo F, Humphries RK, Brock HW (2006). Characterization of Asxl1, a murine homolog of Additional sex combs, and analysis of the Asx-like gene family.. Gene.

[43] Katoh M, Katoh M (2003). Identification and characterization of ASXL2 gene in silico.. Int J Oncol.

[44] Katoh M, Katoh M (2004). Identification and characterization of ASXL3 gene in silico.. Int J Oncol.

[45] Carbuccia N, Murati A, Trouplin V, Brecqueville M, Adelaide J (2009). Mutations of ASXL1 gene in myeloproliferative neoplasms.. Leukemia.

[46] Kawaguchi H, Manabe N, Miyaura C, Chikuda H, Nakamura K (1999). Independent impairment of osteoblast and osteoclast differentiation in klotho mouse exhibiting low-turnover osteopenia.. J Clin Invest.

[47] Kenner L, Hoebertz A, Beil T, Keon N, Karreth F (2004). Mice lacking JunB are osteopenic due to cell-autonomous osteoblast and osteoclast defects.. J Cell Biol.

[48] Kawamura N, Kugimiya F, Oshima Y, Ohba S, Ikeda T (2007). Akt1 in osteoblasts and osteoclasts controls bone remodeling.. PLoS ONE.

[49] Zhao H, Ito Y, Chappel J, Andrews NW, Teitelbaum SL (2008). Synaptotagmin VII regulates bone remodeling by modulating osteoclast and osteoblast secretion.. Dev Cell.

[50] Petkov PM, Graber JH, Churchill GA, DiPetrillo K, King BL (2005). Evidence of a large-scale functional organization of mammalian chromosomes.. PLoS Genet.

[51] Grundberg E, Kwan T, Ge B, Lam KC, Koka V (2009). Population genomics in a disease targeted primary cell model.. Genome Res.

[52] Lin SM, Du P, Huber W, Kibbe WA (2008). Model-based variance-stabilizing transformation for Illumina microarray data.. Nucleic Acids Res.

[53] Du P, Kibbe WA, Lin SM (2008). lumi: a pipeline for processing Illumina microarray.. Bioinformatics.

[54] Johnson WE, Li C, Rabinovic A (2007). Adjusting batch effects in microarray expression data using empirical Bayes methods.. Biostatistics.

[55] Yu J, Pressoir G, Briggs WH, Vroh Bi I, Yamasaki M (2006). A unified mixed-model method for association mapping that accounts for multiple levels of relatedness.. Nat Genet.

[56] Zhao K, Aranzana MJ, Kim S, Lister C, Shindo C (2007). An Arabidopsis example of association mapping in structured samples.. PLoS Genet.

[57] Chen L, Page GP, Mehta T, Feng R, Cui X (2009). Single nucleotide polymorphisms affect both cis- and trans-eQTLs.. Genomics.

[58] Alberts R, Terpstra P, Li Y, Breitling R, Nap JP (2007). Sequence polymorphisms cause many false cis eQTLs.. PLoS ONE.

[59] Langfelder P, Horvath S (2008). WGCNA: an R package for weighted correlation network analysis.. BMC Bioinformatics.

[60] Farber CR (2010). Identification of a gene module associated with BMD through the integration of network analysis and genome-wide association data.. J Bone Miner Res.

[61] Langfelder P, Zhang B, Horvath S (2008). Defining clusters from a hierarchical cluster tree: the Dynamic Tree Cut package for R.. Bioinformatics.

