# Supplementary figures and images for: Mouse Genome-Wide Association and Systems Genetics Identify Asxl2 As a Regulator of Bone Mineral Density and Osteoclastogenesis
Source: PLoS Genet. 2011 Apr 7;7(4):e1002038. doi: 10.1371/journal.pgen.1002038 (PMC3072371; doi:10.1371/journal.pgen.1002038)

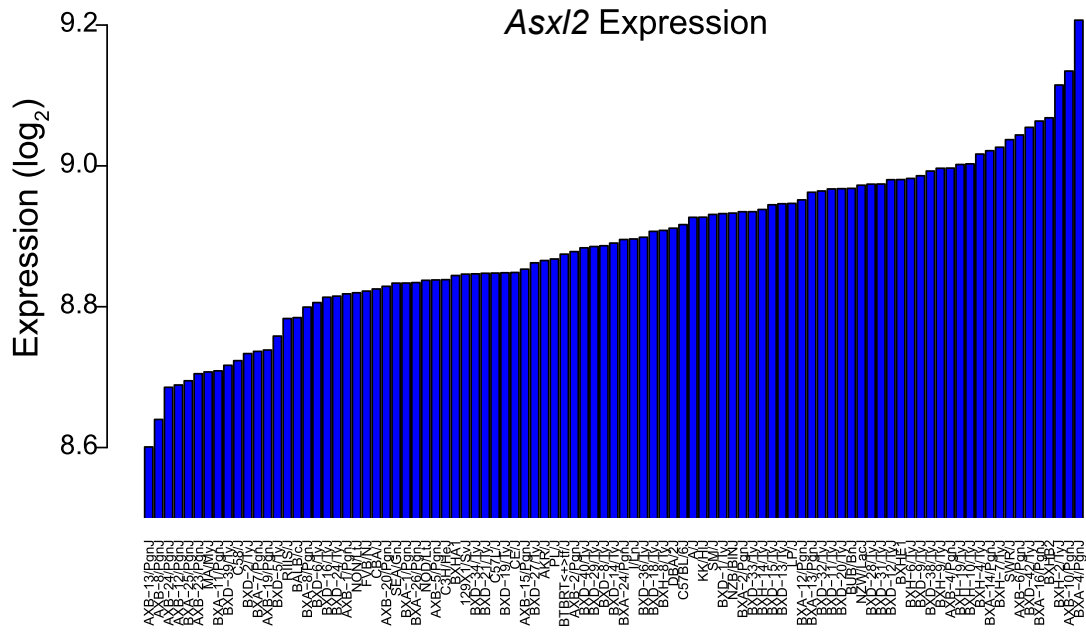

Supplement: Figure S1 — The expression of Asxl2 across HMDP strain is highly variable. Asxl2 expression values from cortical bone (femoral diaphysis with marrow removed) microarray data from 95 HMDP strains. Asxl2 was highly expressed in bone and its expression varies by 1.5-fold (log2 difference of 0.6) in the lowest and highest expressing strains. (PDF) [file pgen.1002038.s001.pdf]
